# Successful Profiling of Plasmodium falciparum *var* Gene Expression in Clinical Samples via a Custom Capture Array

**DOI:** 10.1128/mSystems.00226-21

**Published:** 2021-11-30

**Authors:** Emily M. Stucke, Antoine Dara, Ankit Dwivedi, Theresa K. Hodges, Sandra Ott, Drissa Coulibaly, Abdoulaye K. Koné, Karim Traoré, Bouréima Guindo, Bourama M. Tangara, Amadou Niangaly, Modibo Daou, Issa Diarra, Youssouf Tolo, Mody Sissoko, Luke J. Tallon, Lisa Sadzewicz, Albert E. Zhou, Matthew B. Laurens, Amed Ouattara, Bourema Kouriba, Ogobara K. Doumbo, Shannon Takala-Harrison, David Serre, Christopher V. Plowe, Mahamadou A. Thera, Mark A. Travassos, Joana C. Silva

**Affiliations:** a Malaria Research Program, Center for Vaccine Development and Global Health, University of Maryland School of Medicine, Baltimore, Maryland, USA; b Institute for Genome Sciences, University of Maryland School of Medicine, Baltimore, Maryland, USA; c Malaria Research and Training Center, University of Science, Techniques and Technologies, Bamako, Mali; d Department of Microbiology and Immunology, University of Maryland School of Medicine, Baltimore, Maryland, USA; Tufts University

**Keywords:** *P. falciparum*, malaria, PfEMP1, *var* gene, *Plasmodium falciparum*, RNA-Seq

## Abstract

*var* genes encode Plasmodium falciparum erythrocyte membrane protein-1 (PfEMP1) antigens. These highly diverse antigens are displayed on the surface of infected erythrocytes and play a critical role in immune evasion and sequestration of infected erythrocytes. Studies of *var* expression using non-leukocyte-depleted blood are challenging because of the predominance of host genetic material and lack of conserved *var* segments. Our goal was to enrich for parasite RNA, allowing *de novo* assembly of *var* genes and detection of expressed novel variants. We used two overall approaches: (i) enriching for total mRNA in the sequencing library preparations and (ii) enriching for parasite RNA with a custom capture array based on Roche’s SeqCap EZ enrichment system. The capture array was designed with probes based on the whole 3D7 reference genome and an additional >4,000 full-length *var* gene sequences from other P. falciparum strains. We tested each method on RNA samples from Malian children with severe or uncomplicated malaria infections. All reads mapping to the human genome were removed, the remaining reads were assembled *de novo* into transcripts, and from these, *var*-like transcripts were identified and annotated. The capture array produced the longest maximum length and largest numbers of *var* gene transcripts in each sample, particularly in samples with low parasitemia. Identifying the most-expressed *var* gene sequences in whole-blood clinical samples without the need for extensive processing or generating sample-specific reference genome data is critical for understanding the role of PfEMP1s in malaria pathogenesis.

**IMPORTANCE** Malaria parasites display antigens on the surface of infected red blood cells in the human host that facilitate attachment to blood vessels, contributing to the severity of infection. These antigens are highly variable, allowing the parasite to evade the immune system. Identifying these expressed antigens is critical to understanding the development of severe malarial disease. However, clinical samples contain limited amounts of parasite genetic material, a challenge for sequencing efforts further compounded by the extreme diversity of the parasite surface antigens. We present a method that enriches for these antigen sequences in clinical samples using a custom capture array, requiring minimal processing in the field. While our results are focused on the malaria parasite Plasmodium falciparum, this approach has broad applicability to other highly diverse antigens from other parasites and pathogens such as those that cause giardiasis and leishmaniasis.

## INTRODUCTION

While there has been an overall decrease in the number of malaria cases and deaths in the last decade, these numbers have plateaued since 2015 (1). More than 90% of malaria deaths occur in sub-Saharan Africa (1), primarily the result of the most severe forms of malaria caused by Plasmodium falciparum, including cerebral malaria (CM) and severe malarial anemia (SMA). Protection against malaria illness can be acquired over time in regions of endemicity, likely as a repertoire of immune responses to parasite variant surface antigens (VSAs) displayed on the surface of infected erythrocytes. Protection from clinical malaria has been associated with the presence of antibodies to these particular antigens (2–4). In P. falciparum, VSAs are encoded by three main multigene families: *var*, *rif*, and *stevor* genes.

The most widely studied VSAs are the P. falciparum erythrocyte membrane protein-1 (PfEMP1) antigens, which are encoded by the *var* gene family. Approximately 60 PfEMP1s are encoded by *var* genes in each P. falciparum genome (5, 6), and can be classified into different groups (A, B, C, and E) using the upstream promoter sequence (7). These are highly diverse antigens displayed on the surface of infected erythrocytes, and they play a critical role in immune evasion and sequestration. PfEMP1 antigens are composed of combinations of domains that include two to eight Duffy binding-like (DBL) domains and one to two cysteine-rich interdomain regions (CIDRs). Each infected erythrocyte displays on its surface only one particular PfEMP1 (8), which mediates binding to different host cell receptors (9, 10).

Determining PfEMP1s that are specific to different severe malaria syndromes is essential for understanding the pathogenesis of severe malaria. Studies in both adults and children have associated PfEMP1 subtypes and *var* groups with severe malaria syndromes, with conflicting results that suggest that some similar subtypes are associated with both cerebral malaria and severe malarial anemia. Domain cassettes (DCs), or consecutive DBL and CIDR domains found to recur across multiple PfEMP1s, have been associated with severe malarial disease. DC8 (11–14), DC13 (11, 13), and group A *var* genes (11, 14–17) have all been associated with severe malaria syndromes. These studies have primarily used quantitative reverse transcription PCR (qRT-PCR), targeting usually a domain or domain cassette with primers designed against a subset of known sequences and comparing the expression between groups of participants with severe malarial disease and controls with uncomplicated malarial disease. Domains or *var* gene groups with significantly greater transcript levels were associated with severe malaria syndromes, but these methods did not identify the most highly expressed *var* gene in an infection. Determining the most-expressed *var* gene in an infection, including identification of novel *var* gene sequences, requires an unbiased approach that allows detection of any independent *var* transcript present.

Sequencing full-length *var* genes from clinical samples has proven challenging. PfEMP1s often have <50% shared amino acid identity, and the full *var* repertoires are known for only a small number of reference genomes and clinical samples (5, 6, 18–20). The average shared amino acid identity is ∼30 to 40% within classes of DBL and CIDR domains (18), indicating that diverse sequences occur even within classes of domains. This diversity and lack of recurring motifs add to the challenge of sequencing these genes. The variable length of *var* genes is another challenge, ranging from ∼4,500 to ∼12,000 bp and up to 2 orders of magnitude longer than the standard sequencing read of 150 bp. Each P. falciparum genome contains multiple copies of *var* genes—usually ∼60—featuring an internal repeated motif structure that makes assembly difficult. Studies of *var* gene expression have focused on a degenerate primer approach, capturing only a few domains at a time (11–14, 21), which does not enable a comparison of the entire *var* gene sequence. Another challenge in sequencing *var* genes is the relatively small proportion of *var* transcripts compared to host and the total parasite transcriptome, particularly in samples collected as whole blood. Clinical samples collected as whole blood are composed of mostly host (human) RNA and DNA, with parasite genetic material composing a smaller proportion. A recent study successfully assembled *var* transcripts *de novo* from clinical malaria samples in Indonesia; however, the methods required leukocyte-depleted blood (22).

An effective method to identify and sequence highly expressed genes encoding variable antigens has the potential to provide insight into immune evasion and the pathogenesis of specific clinical syndromes for a range of pathogens, including P. falciparum, Plasmodium vivax, Giardia, and *Leishmania*. Here, we attempt to overcome existing challenges in generating a comprehensive profile of the expressed *var* genes and determining their relative expression in specific severe malaria phenotypes by applying different approaches to enrich parasite RNA in a sample and applying these methods to total RNA extracted from whole-blood clinical samples. We compared two overall approaches: (i) capture of parasite RNA, using a capture array designed from the 3D7 genome (excluding the ribosomal DNA [rDNA] loci) plus >4,000 *var* gene sequences from other P. falciparum strains (designated Capture), and (ii) two methods of depleting undesired RNA types, depletion of rRNA and globin mRNA (designated Depletion) and depletion of rRNA and globin mRNA followed by selection for polyadenylated transcripts [designated Depletion + poly(A)].

We recently sequenced and assembled full *var* repertoires from genomic sequence data from uncomplicated malaria infections in Malian children (19). As proof of principle, in the present study we applied the parasite RNA enrichment methods to 11 RNA samples from those same uncomplicated malaria infections, for which genomic data and complete, reconstructed *var* repertoires were available for reference. We evaluated the outcome of those methods and our protocol for *de novo* assembly of expressed *var* genes using as a reference the genomic *var* gene repertoires. We then applied our methods to a subset of samples from a case-control study of severe malaria, including cases with cerebral malaria or severe malarial anemia. We describe parasite RNA enrichment from whole-blood clinical samples and successful sequencing and assembly of expressed *var* genes using a custom capture approach, which is particularly useful for samples from low-parasitemia infections. While our results are focused on P. falciparum, this approach has broad applicability to other highly diverse antigens from a variety of parasites and pathogens such as those that cause giardiasis and leishmaniasis.

## RESULTS

### Sequencing data generated from each enrichment method.

**FIG 1:**
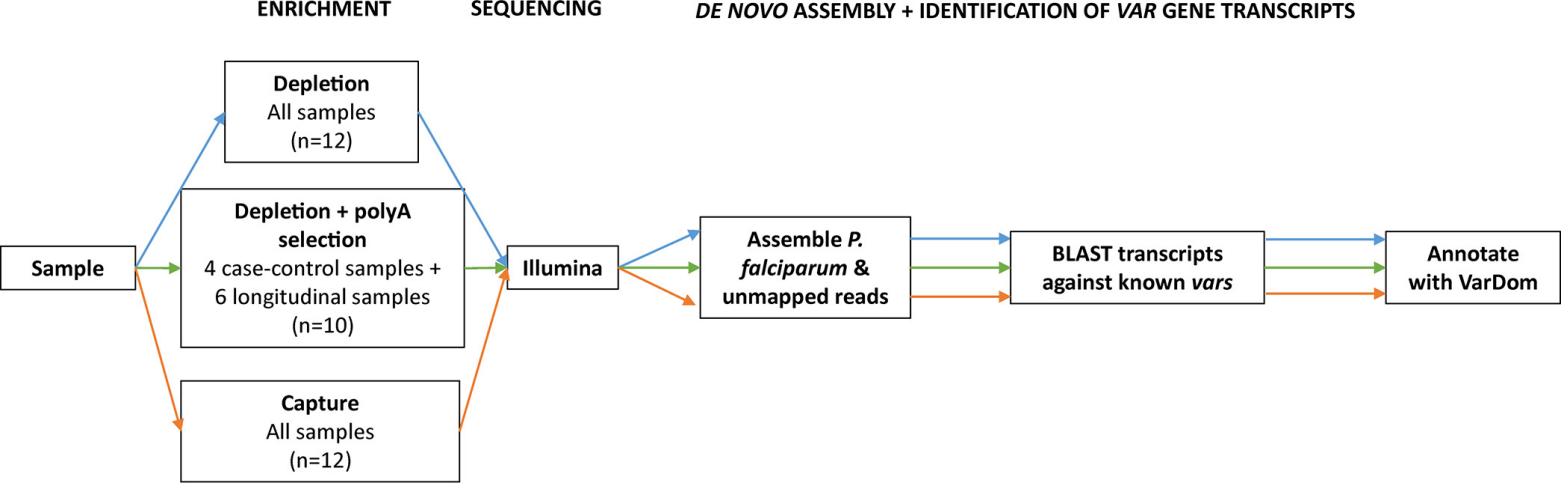
Overview of pipeline for enrichment of parasite RNA, sequencing, and assembly of transcripts. P. falciparum mRNA was enriched in all 12 samples using both globin and rRNA depletion as well as capture. Ten samples were subjected to all three methods, including globin and rRNA depletion followed by poly(A) selection. For the Depletion and Depletion + poly(A) method, enrichment took place during library preparation, and for Capture, libraries were prepared, and then Capture was applied to enrich for parasite cDNA. Reads were mapped to a concatenated reference of human and P. falciparum. We used reads that mapped to P. falciparum and unmapped reads to *de novo* assemble transcripts, open reading frames for the transcripts were subjected to a protein blast search to identify *var* transcripts, and then the protein sequences were annotated with the VarDom online database.

To determine the performance of different methods to enrich P. falciparum mRNA from a sample of total RNA before sequencing, we used a total of 12 samples, four from a case-control study and eight from a longitudinal study. All four samples from the case-control study were used to evaluate each method of enrichment: Depletion, Depletion + poly(A) selection, and Capture (Table 1, Fig. 1). All longitudinal study samples (*n* = 8) were used for Depletion and Capture, while six were used for Depletion + poly(A) selection [no Depletion + poly(A) data for samples UM 2 and UM 5 (Table 1)]. The average number of total reads sequenced was 83.9 million for the Depletion method, 72.7 million for Depletion + poly(A), and 73.2 million for Capture (Table 1; see also Table S1 and Fig. S1 in the supplemental material). Reads mapping to P. falciparum and unmapped reads were more variable, averaging 19.7 million reads for Depletion method, 15.8 million reads for Depletion + poly(A), and 72.1 million reads for Capture (Table S1 and Fig. S1).

**TABLE 1:** Characteristics of samples for enrichment of parasite RNA and the enrichment methods applied to each sample

Sample	Study source	Parasitemia (no. of parasites/μl)	Enrichment methods (total reads)		
			Depletion	Depletion + poly(A)	Capture
CM + SMA	Case-control	1,650	56,722,984	74,499,556	86,791,160
SMA	Case-control	143,750	70,337,818	69,473,914	66,787,024
UMC	Case-control	14,700	63,480,128	52,348,806	82,076,018
CM	Case-control	11,680	73,356,546	68,572,664	75,814,008
UM 1	Longitudinal	209,050	85,943,726	88,712,300	83,561,130
UM 2	Longitudinal	15,400	94,326,578		85,030,980
UM 3	Longitudinal	185,400	94,676,230	67,703,494	42,798,838
UM 5	Longitudinal	7,525	85,612,460		50,651,528
UM 6	Longitudinal	193,800	115,500,792	79,058,422	44,708,486
UM 8	Longitudinal	19,275	91,193,310	70,058,484	48,861,952
UM 9	Longitudinal	198,900	92,401,680	72,452,038	120,660,408
UM 11	Longitudinal	14,650	83,334,470	84,384,254	90,965,320

10.1128/mSystems.00226-21.1 FIG S1 Comparison of the number of 20 million randomly selected reads mapping to known *var* genes from each sample for each library preparation. The total number of randomly selected reads from Capture (orange) that mapped to known *var* genes was significantly greater than Depletion (blue) and Depletion + poly(A) (green) (Wilcoxon signed-rank test). Download FIG S1, PDF file, 0.04 MB. Copyright © 2021 Stucke et al. 2021 Stucke et al. https://creativecommons.org/licenses/by/4.0/ This content is distributed under the terms of the Creative Commons Attribution 4.0 International license.

10.1128/mSystems.00226-21.5 TABLE S1 Sample information, total reads, Plasmodium falciparum reads, insert size, and read length. Download Table S1, XLSX file, 0.01 MB. Copyright © 2021 Stucke et al. 2021 Stucke et al. https://creativecommons.org/licenses/by/4.0/ This content is distributed under the terms of the Creative Commons Attribution 4.0 International license.

### Capture yields a consistently greater proportion of reads from parasite transcripts.

**FIG 2:**
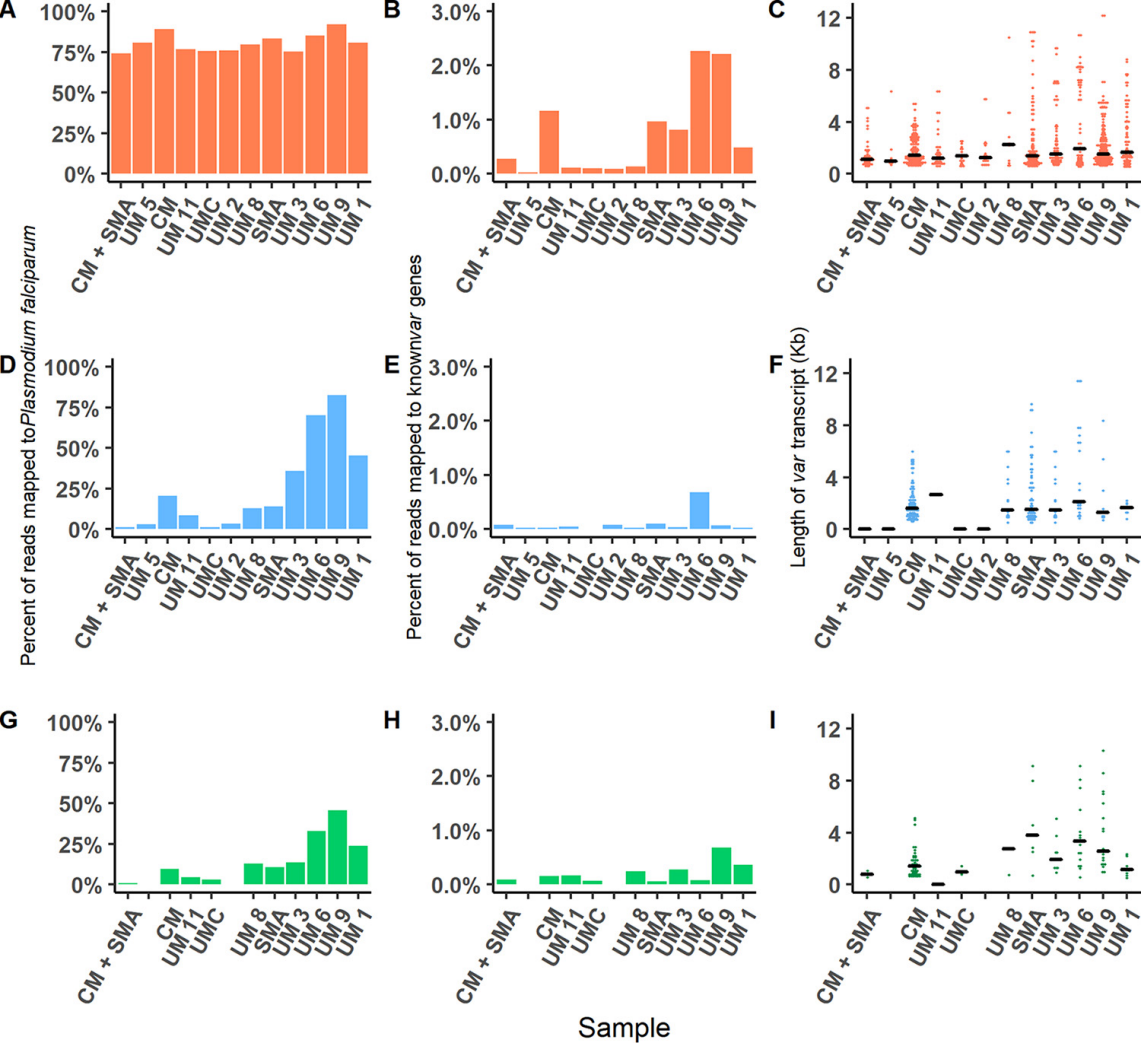
Reads mapping to P. falciparum, known *var* genes, and number and length of assembled *var* gene transcripts. Results were quantified for Capture (orange; first row), Depletion (blue; second row), and Depletion + poly(A) (green; third row). Reads mapping to P. falciparum were quantified for the three methods (A, D, and G); percentages of the random selection of 20 million reads mapping to known *var* genes were quantified for the three methods (B, E, and H); and the number and length of unique transcripts were quantified for the three methods (C, F, and I). Capture retained the greatest percentages of P. falciparum and *var* reads, and more unique *var* transcripts were assembled from the Capture-prepared samples. Samples are arranged from least (left) to greatest parasitemia. CM, cerebral malaria; UM, uncomplicated malaria; UMC, uncomplicated malaria control; SMA, severe malarial anemia.

As a preliminary measure of enrichment, we first quantified the number and proportions of sequence reads that mapped to the P. falciparum reference genome to determine which library preparation most consistently recovers the greatest amount and proportion of parasite reads (Fig. 2A, D, and G). Reads mapping to the P. falciparum genome were identified from all libraries and were a greater proportion of the total reads from the Capture libraries than from the Depletion libraries. This outcome is to be expected, inasmuch as the Capture approach enriches parasite nucleic acids specifically by capturing cDNA with sequences similar to the 3D7 genome. However, we also observed a more consistent percentage of reads mapping to the P. falciparum genome from samples prepared with Capture enrichment, with a mean of 81% (range, 75 to 90%) (Fig. 2A). In contrast, for the Depletion and Depletion + poly(A) libraries, we observed a wider range of percentages mapping to the P. falciparum genome (<5% to 80%), which was positively associated with parasitemia levels. For the Depletion libraries, a mean of 23% of reads mapped to P. falciparum, with ∼1% of reads mapped to P. falciparum in the CM + SMA sample with low parasitemia (1,650 parasites/μl), versus around 80% of mapped reads in UM 6 and UM 9 with a higher parasitemia of ∼200,000 parasites/μl (Fig. 2D). For the Depletion + poly(A) libraries, we calculated a mean of 15% of reads mapped to P. falciparum, ranging from <1% in the lowest-parasitemia sample CM + SMA to ∼50% in sample UM 9 with ∼200,000 parasites/μl (Fig. 2G).

### Capture produces greater percentages of *var* reads and assembled *var* transcripts.

To determine which preparation produced the greatest proportion of reads mapping to *var* genes, we selected a random sample of 20 million paired reads from each library to normalize by data set size to the smallest data set sequenced. These read subsets were mapped to an index of >4,000 *var* gene sequences from lab strains, reference genomes, and clinical samples (described in Materials and Methods) to determine the percentage of reads mapping to known *var* genes (Fig. 2B, E, and H). Fewer than 1% of reads mapped to the index of known *var* genes from the Depletion and Depletion + poly(A) libraries, which contrasts with Capture libraries in which reads mapping to *var* genes were identified from every sample. Capture produced greater percentages of *var* reads, which surpassed 1% for three samples (CM, UM 6, and UM 9) (Fig. 2B). Using the 20 million randomly selected reads, Capture libraries had a significantly greater median number of reads mapping to known *var* genes than Depletion libraries (Wilcoxon signed-rank test, *P* < 0.001 [Fig. S2]) and Depletion + poly(A) libraries (Wilcoxon signed-rank test, *P* < 0.05). The Capture approach thus produced more reads, and reads from the total library that were then assembled into more transcripts for each sample than the other library preparation methods (Fig. 2C).

10.1128/mSystems.00226-21.2 FIG S2 Total number of reads mapping to the P. falciparum reference genome or unmapped (red) or the human reference genome (blue) for each library preparation [Capture, left; Depletion, center; Depletion + poly(A), right]. Reads mapping to P. falciparum or unmapped are a larger proportion of total reads in the Capture (left) library preparation than in those of Depletion (center) and Depletion + poly(A) (right). Samples are arranged from least to greatest parasitemia, left to right. Download FIG S2, PDF file, 0.03 MB. Copyright © 2021 Stucke et al. 2021 Stucke et al. https://creativecommons.org/licenses/by/4.0/ This content is distributed under the terms of the Creative Commons Attribution 4.0 International license.

We compared the use of two different references to identify *var* transcripts from the reconstructed transcriptome, by using (i) as reference *var* sequences from the 3D7 reference genome versus (ii) an extended set of known *var* sequences from several reference genomes and clinical isolates included in the design of the capture array. For Capture, we were able to identify all transcripts with PfEMP1 domains using either reference set. For Depletion + poly(A), we identified 95% of transcripts with PfEMP1 domains using only the 3D7 reference. While the larger database strategy matched sequences with greater amino acid identity, the transcripts with *var* domains matched 3D7 *var* sequences with sufficiently high amino acid identity to be recognized.

To compare the assemblies of expressed *var* genes from each library, we quantified the number of *de novo*-assembled *var* transcripts and determined their length. We were able to assemble transcripts with one or more domains from all libraries prepared with Capture (Fig. 2C); however, for Depletion and Depletion + poly(A) libraries for samples with lower parasitemias (parasitemia of <15,000/μl) we assembled very few or no transcripts (Fig. 2F and I). This included no transcripts for four samples using the Depletion library preparation (CM + SMA, UM 5, UMC [uncomplicated malaria control], and UM 2) and for one sample using the Depletion + poly(A) preparation (UM 11) (Fig. 2F). We were able to assemble multiple transcripts from sample CM using each of the enrichment methods, but the percentage of reads and the number of transcripts were improved with Capture. Overall, Capture produced greater percentages of reads mapping to *var* genes and more *de novo*-assembled *var* transcripts.

### Enrichment methods yield similar results for most-expressed *var* genes.

**FIG 3:**
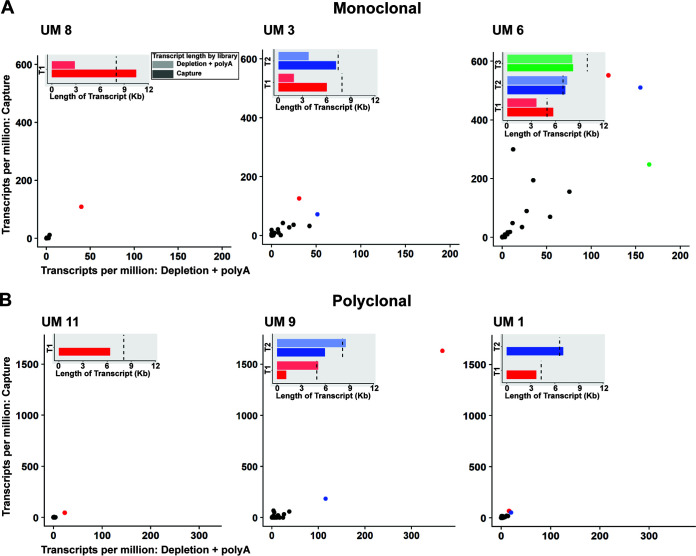
Comparison of expression and assembly results from Capture and Depletion + poly(A) mapping to genomic sequences. Each scatterplot compares transcripts per million (TPMs) in Depletion + poly(A) on the *x* axis to Capture on the *y* axis by mapping the reads from each library to the genomic reference. The bar graph in the inset of each panel shows the length of the most-expressed transcripts *de novo* assembled from the RNA-Seq data [Capture, darker shade; Depletion + poly(A), lighter shade] and the length of the corresponding genomic sequence (dotted black line). For each uncomplicated malaria sample, the same *var* genes were identified in the two libraries, but Capture yielded greater transcripts per million (scatterplots). Predominantly expressed *var* genes *de novo* assembled from RNA-Seq data were similar in length (bar graphs) to the genome reference *var* genes (dotted black line), with *de novo*-assembled sequences from capture typically longer than those from Depletion + poly(A). Panel A (UM 8, UM 3, and UM 6) includes monoclonal (one infecting parasite clone) samples, and panel B includes polyclonal (more than one infecting parasite clone) samples (UM 11, UM 9, and UM 1). Samples are arranged from least to greatest parasitemia within each row, from left to right. The color of the dots in the scatterplot (red, blue, or green) corresponds to the same transcript (T1, T2, or T3) shown assembled in the inset bar graph. UM, uncomplicated malaria; T1, transcript 1; T2, transcript 2; T3, transcript 3.

As proof of principle for our assembly and quantification methods, namely, to confirm that we are reconstructing *var* genes of the correct length, not chimeras, and that reconstructed transcripts can be used to quantify expression, we focused on the longitudinal study samples with corresponding genomic data (UM 1, UM 2, UM 3, UM 8, UM 9, and UM 11). To determine which library preparation yields the most reliable relative quantification of *var* gene expression and if expression levels were similar between the Depletion + poly(A) and Capture enrichment methods, we mapped reads to the genomic *var* sequences from each sample (Fig. 3). For each uncomplicated malaria sample with a genomic *var* reference, the same *var* genes were detected in both libraries, but with greater transcripts per million (TPMs) in those from Capture. For samples UM 8, UM 11, and UM 9, there was a single most-expressed *var* gene identified by both enrichment methods. In samples UM 3 and UM 1, the top two most-expressed *var* genes were the same but the order of expression magnitude differed. For sample UM 6, the most-expressed *var* gene was the same, but the order of the second and third most-expressed differed between Capture and Depletion + poly(A). For the case-control samples with no genomic reference, we compared *var* gene expression levels by mapping the reads from each library separately to the reference using a common *de novo var* reference assembled with reads from both Capture and Depletion + poly(A) libraries (Fig. S3). We detected the same *var* genes from both libraries, and no *var* genes detected by Depletion + poly(A) were not detected by Capture, indicating that the use of Capture probes did not result in a failure to detect particular transcripts.

10.1128/mSystems.00226-21.3 FIG S3 Comparison of transcripts per million for expressed *var* genes in Depletion + poly(A) and Capture. TPMs shown in scatterplots are for the *var* genes expressed in the four case-control study samples. TPMs are calculated from mapping to a common reference *de novo* assembled by combining reads from both Depletion + poly(A) and Capture libraries. Capture identified the same expressed *var* genes in the case-control samples as did Depletion + poly(A), but with more transcripts per million. CM, cerebral malaria; UMC, uncomplicated malaria control; SMA, severe malarial anemia. Download FIG S3, PDF file, 0.02 MB. Copyright © 2021 Stucke et al. 2021 Stucke et al. https://creativecommons.org/licenses/by/4.0/ This content is distributed under the terms of the Creative Commons Attribution 4.0 International license.

We next determined if we were able to assemble the sequences of the most-expressed *var* genes from the RNA sequencing (RNA-Seq) data. For each sample, we identified sequences from the transcriptome sequencing *de novo* assembly matching with >99% identity to the genomic *var* sequences (Fig. 3, inset). Predominantly expressed *var* genes were similar in length to the genomic sequences (Fig. 3, dotted black lines in insets) and most commonly assembled with Capture. However, we were not able to assemble sequences matching the most-expressed *var* genes from two samples—UM 11 and UM 1—with the Depletion + poly(A) method. Depletion + poly(A) worked particularly well for sample UM 9, and while we identified matching sequences with Capture, the sequences were not the same length as the genomic sequence.

### Most-expressed *var* genes were similar whether using genomic or *de novo*-assembled sequences as reference.

Unlike the present study, most *var* transcription studies using field samples lack reference whole-genome sequences, and quantification of expression will need to be done using *de novo*-assembled transcripts as reference. To determine the reliability of this approach, we identified the most-expressed *var* genes from mapping the RNA-Seq reads to both the genomic reference as well as to the *var* transcripts assembled *de novo* from the RNA-Seq data. Using data generated from Capture, we found that 5/8 samples had the same most-expressed *var* gene with mapping to either the genomic or *de novo* transcripts (UM 3, UM 5, UM 8, UM 9, and UM 11 [Fig. S4]). In an additional two samples, the same *var* genes were the top two most expressed; however, the order of expression varied for each reference (UM 1 and UM 6 [Fig. S4]).

10.1128/mSystems.00226-21.4 FIG S4 Proportions of *var* gene expression for Capture mapping to *de novo*-assembled sequences and to genomic sequences. The most-expressed *var* gene was the same mapping to the *de novo* and to the genomic references for samples UM 3, UM 5, UM 8, UM 9, and UM 11. Each color represents a distinct *var* sequence within each sample, except for the checkered wedges, which represent *var* sequences with expression of <5% of the total *var* expression. Wedges that are different pieces but the same color within a *de novo* reference pie correspond to two different parts of the same genomic sequence (only within the same sample); however, the sequences assembled as two different distinct sequences in the *de novo* assembly with rnaSPAdes. UM, uncomplicated malaria. Download FIG S4, PDF file, 0.2 MB. Copyright © 2021 Stucke et al. 2021 Stucke et al. https://creativecommons.org/licenses/by/4.0/ This content is distributed under the terms of the Creative Commons Attribution 4.0 International license.

## DISCUSSION

To understand the impact of PfEMP1 in malaria pathogenesis, it is essential to determine which *var* genes are expressed in an infection and which transcript(s) is the most abundant. Sequencing expressed *var* genes from clinical samples has proven difficult owing to the diversity and motif repetition of the gene family. Achieving those goals with a single conserved set of primers to amplify all *var* genes has not been possible because of the extreme variation and the large numbers of *var* genes per genome. Another difficulty in identifying *var* transcripts from clinical samples is obtaining adequate parasite RNA for sequencing as almost all RNA in these specimens belongs to the human host. These obstacles pose significant challenges to understanding expression of *var* genes in clinical samples. Therefore, the goal of this work was to determine the viability of mRNA and parasite RNA enrichment methods to recover this information.

One approach, enriching for mRNA during library preparation [Depletion and Depletion + poly(A)], was intended to enrich for parasite mRNA in the sample by eliminating the two most abundant transcripts in blood samples, namely, rRNA, which altogether constitutes over ∼90% of all host RNA, and globin RNA, the next most abundant transcript in human blood (23). The potential pitfall of this method is that it also enriches for human mRNA. An alternative approach available to us was to enrich specifically for all parasite RNA using capture probes based on the 3D7 reference genome supplemented with additional sequences of the variant surface antigens PfEMP1s, RIFINs, and STEVORs. Potential pitfalls of the capture approach were that our probe set included probes for the entire genome sequence (albeit enriched for variant surface antigens) and that *var* transcript detection depended on hybridization to the (potentially quite different) reference *var* sequences used in probe design. To evaluate our assembly methods and the Capture approach, we used two sets of samples. One set was used for proof of principle, since the corresponding genome assembly was available and allowed us to determine the accuracy of the reconstructed *var* transcripts, as well as to determine if their relative abundance matches would be expected from mapping the reads to the genome. With the second set of samples, we aimed to determine the feasibility of this overall study with samples from a case-control study for which no reference genome data were available. The proof-of-principle approach showed that the *var* transcripts were reconstructed accurately from the RNA-Seq reads obtained. This is supported by a study that reconstructed genomic *var* gene sequences from whole-genome sequencing data for >2,000 samples (5). In addition, the proof-of-principle samples showed that the Capture approach resulted in an increased proportion of *var* reads relative to the Depletion methods and that the reconstructed transcriptome can serve as a reference to identify the most abundantly transcribed *var* gene. The application of these approaches to the case-control study suggests that, at least with this limited number of samples, the Capture-based method did not result in missed *var* transcripts, since the Depletion methods did not reveal novel *var* transcripts that were not also identified with Capture.

Using the SeqCap enrichment system with custom capture probes, we were able to sequence mostly P. falciparum RNA and retain a greater proportion of *var*-like reads compared with depletion of globin mRNA and rRNA transcripts or with depletion of globin mRNA and rRNA transcripts followed by selection of polyadenylated transcripts. In addition, this method produced more unique transcripts, including transcripts from low-parasitemia samples that were not recovered either with depletion of globin mRNA and rRNA transcripts or with depletion of globin mRNA and rRNA transcripts followed by selection of polyadenylated transcripts. Interestingly, in the case-control samples, we observed greater percentages of reads mapping to *var* genes in the severe malaria samples from Capture than from the other enrichment methods, even for samples with lower parasitemia. This could indicate that parasites from severe malaria infections are producing more *var* transcripts than those from uncomplicated malaria infections.

Assembly and reconstruction of *var* genes from clinical samples have been successful using genomic sequencing data (5, 6, 19), and one recent study has successfully assembled *var* genes from RNA sequencing of clinical samples (22). A published study presented *var* gene repertoires assembled from >2,000 clinical isolates from 12 countries (5), *de novo* assembling *var* genes from Illumina sequence data from reads mapping to the 3D7 reference genome. The completeness of each *var* complement was assessed by quantifying a conserved motif in the first domain of most *var* genes, DBLα, and by limiting the quantified *var* genes to those of >3 kb. The *de novo* assemblies of *var* genes were confirmed by comparison to whole-genome assemblies of 15 field isolates with PacBio sequencing (6). While PacBio sequencing has the advantage of longer reads that will sequence through the entire length of a variant surface antigen, this technology requires a large amount of genetic material that is usually generated from short-term parasite cultures. One study thus far has assembled *var* genes to quantify *var* gene expression in clinical samples using Illumina sequencing data. Similar to our approach, a study of severe and uncomplicated malaria with Indonesian samples used *de novo* assembly methods to assemble *var* gene transcripts from RNA sequencing data (22). However, this method for RNA sequencing was limited to leukocyte-depleted blood, which requires a time-consuming process that must be conducted in the field at sample collection. In contrast, we employ a Capture method that can be applied on a large scale without extensive sample processing in the field to understand how *var* expression, defined in terms of transcripts with multiple domains, is associated with severe malaria. Furthermore, we were able to evaluate and validate our assembly methods by comparison with whole-genome sequencing data generated from samples collected at the same time.

Our study did have some limitations. We included only a small subset of samples from a case-control study of severe malaria and thus cannot draw any definitive conclusions about the nature of transcription in severe malaria samples. We were also limited in testing different enrichment methods on all included samples by the amount of RNA that we were able to isolate. The amount of RNA was sufficient to employ all three of our enrichment methods on the subset of case-control samples. However, we had adequate RNA to test only two enrichment methods on six samples of the 11 uncomplicated malaria samples from the longitudinal study and only one method for the remaining five.

The Capture method may also have some limitations. It is possible that enrichment with Capture may produce some bias toward the expression of particular *var* genes, but we did not find evidence of this in comparing expression methods. Expression using different methods for the same sample was generally in agreement in terms of the most-expressed *var* sequences. We did not find any *var* genes highly expressed in the Depletion + poly(A) preparation that were not observed with Capture. The broad concordance between the two methods indicates that we are able to identity the most-expressed *var* gene. However, the rank order of *var* expression varied in the situation when the expression levels of particular *var* genes were close in magnitude. The results from Capture would benefit from confirmation with other methods to study expression, such as qRT-PCR to confirm the most-expressed *var* gene. Finally, capture approaches can be expensive relative to leukocyte depletion, representing a tradeoff between cost and sample processing time in the field. Capture, costing an additional ∼$1,000 per sample, produced on average 3 to 4 times more reads mapping to P. falciparum than did Depletion and Depletion + poly(A) at an additional $125 per sample. To generate the same number of P. falciparum reads, the libraries from samples prepared using Depletion or Depletion + poly(A) would have to be sequenced 3 to 4 times more, thereby tripling or quadrupling the sequencing cost of these enrichment methods, an increase of several hundreds of dollars. In addition, the cost of Capture for these results was estimated based on a singleplex reaction (single capture per reaction), and multiplexing 3 to 4 samples in the Capture reaction could reduce cost by an added ∼$250 to 300 per sample. While we have established the feasibility of multiplexing genome samples, we are now assessing whether multiplexing RNA samples significantly impacts the dynamic range of transcript abundance estimation.

Enrichment of parasite RNA from whole-blood clinical samples via Capture allowed the successful application of a *de novo* transcript assembly approach for the discovery of novel *var* transcripts, ideal for a diverse gene family. We identified a method to enrich for parasite RNA using Roche’s SeqCap EZ enrichment system with custom capture probes (Capture) to sequence constitutive domains of the expressed *var* genes. This efficient method does not require leukocyte depletion at collection. This comprehensive probe set targets the whole-parasite genome, hence enabling a multitude of applications. However, given the success of this approach, studies that focus exclusively on *var* gene expression could contemplate using a probe set that targets only *var* genes. This method produces a wealth of information from the sequence data of the expressed *var* genes from clinical samples, making possible an analysis of *var* gene expression at multiple levels, including transcript, constitutive domains, domain cassettes, and motifs. Generating these data on a large scale from an observational study without the need to generate whole-genome sequence data facilitates understanding the role of PfEMP1s in malaria pathogenesis. There are aspects of PfEMP1 biology that have not been addressed due to feasibility. Before we can make biological inferences, we must conduct studies on a larger scale that also would allow for the detection of novel variants. This requires an investment in methods that allows for the collection of samples from a large-scale study that can be subjected to RNA sequencing. Additional applications beyond *var* genes in P. falciparum include antigen families in other malaria parasite species such as *vir* genes in P. vivax, as well as highly diverse antigens in other parasites such as those that cause giardiasis and leishmaniasis.

## MATERIALS AND METHODS

### Sample collection.

A total of 2.5 ml of whole blood was obtained from subjects in a case-control study of severe malaria in children in Mali, West Africa, including three children with severe malaria and one uncomplicated malaria control (UMC). The three severe malaria cases included one case of cerebral malaria (CM), one case of severe malarial anemia (SMA), and one case of both cerebral malaria and severe malarial anemia (CM + SMA). Cerebral malaria was defined using the following criteria: Blantyre coma score of ≤2, no other apparent cause of coma, and confirmation of cerebral malaria by malarial retinopathy, an indicator of infected erythrocytes sequestered in the microvasculature of the brain (24). Severe malarial anemia was defined as malarial illness with a hemoglobin level of ≤5 g/dl. A total of 2.5 ml of whole blood was also collected from 11 uncomplicated, symptomatic malaria (UM 1 to 3, 5, 6, 8, 9, and 11 [Table 1]) episodes as part of a longitudinal study of malaria incidence at a vaccine testing site in Bandiagara, Mali (25). For these same 11 samples, both Illumina and Pacific Biosciences (PacBio) whole-genome sequence data were used to reconstruct *var* gene repertoires for each sample (19).

The protocols for the case-control study (HP-00075140) and the longitudinal study of malaria incidence (HP-00041382) were approved by the institutional review boards of the Faculty of Medicine, Pharmacy and Dentistry, Bamako, Mali (FWA00001769), and the University of Maryland School of Medicine (FWA00007145). The consent form was approved by the Institutional Review Board of the Faculty of Medicine, Pharmacy and Dentistry, Bamako, Mali. Written informed consent was obtained from parents or guardians of all study participants, and the study was performed in accordance with the Declaration of Helsinki. All methods were performed in accordance with the relevant guidelines and regulations. Participants consented to publication of study results provided that they were identified only by participant number.

Whole venous blood was preserved in PreAnalytiX PAXgene blood RNA tubes (Qiagen/BD), which were inverted 10 times, stored at room temperature for 2 to 72 h, and then frozen for storage at −80°C according to the manufacturer’s instructions. RNA was extracted with the PreAnalytiX PAXgene blood RNA kit according to the manufacturer’s instructions after thawing the tubes and bringing the tubes to room temperature before extraction. Before processing the RNA for sequencing, samples were evaluated for quality with an Agilent Bioanalyzer to determine the RNA integrity number (RIN), a ratio of the 28S to 18S rRNA, which is an indicator of the sample quality.

### Enrichment methods.

Our first approach was to enrich for parasite RNA with Roche’s SeqCap EZ enrichment system (“Capture”). Biotinylated probes were designed in conjunction with Roche by providing sequences consisting of the 3D7 P. falciparum genome (PlasmoDB v24) with rRNA-encoding genes removed, as well as 2,885 *rif* and *stevor* sequences extracted from clinical isolates, and 4,695 *var* sequences from additional lab strains (DD2, RAJ116, IGH-CR14, 7G8, NF135, and NF166) (7, 18, 20) and clinical isolates from West Africa, East Africa, and Southeast Asia. Probes with lengths between 50 and 150 bp were designed by Roche, using a proprietary algorithm. Probes bound to SeqCap EZ capture beads were then hybridized in solution with the RNA-Seq library, resulting in enrichment of parasite RNA sequences. Unbound sequences were washed away, and the remaining cDNA was amplified and then sequenced using an Illumina HiSeq 4000 platform.

Two methods were used for the second approach of enriching for mRNA: depletion of human globin mRNA and depletion of host and parasite rRNA ((which we refer to, here, as “Depletion”) (Globin-Zero Gold kit; Illumina) and globin mRNA and rRNA depletion followed by poly(A) selection [here “Depletion + poly(A)”] [Ribo-Zero Gold rRNA removal kit (Epidemiology) (Illumina) and NEBNext poly(A) mRNA magnetic isolation module (New England BioLabs)].

Globin mRNA and rRNA were removed with probes that hybridize to rRNA and globin mRNA, leaving mRNA and regulatory RNAs, which are included in the RNA preparation. After enrichment using globin and rRNA depletion, cDNA was generated and libraries with an insert size of 300 to 500 bp were prepared for sequencing with the TruSeq stranded mRNA library prep kit (Illumina) and then sequenced using the Illumina HiSeq 4000.

### RNA-Seq pipeline.

After sequencing, raw data were evaluated for quality by examining the FastQC files, and sequence reads were trimmed if necessary. Using the Eukaryotic RNASeq pipeline at the Institute for Genome Sciences at the University of Maryland School of Medicine, the reads were mapped to a concatenated reference composed of the reference genomes for human (GRCh37) and P. falciparum (PlasmoDB v24) using HISAT2 (v2.0.4) (26). At this step, we determined the percentage of reads mapping to either the human genome or the P. falciparum genome. Using the BAM files converted from SAM files, the files were sorted using SAMtools (v0.1.19) (27), and then seqtk (v1.0) (https://github.com/lh3/seqtk) was used to subset unmapped reads and reads mapping to P. falciparum from the fastq file.

To estimate the number of reads mapping to *var* genes, we constructed an index of 4,695 available *var* gene sequences. From each library, we used seqtk (v1.0) to select a random sample of 20 million paired reads from each library to normalize to the smallest library size and mapped the sample of reads to the known *var* genes to determine the overall alignment rate to known *var* genes. Using a random selection of reads allowed us to compare reads from each library without bias from larger libraries that may have more *var* reads just because more of the sample was sequenced. We used the Wilcoxon signed-rank test to compare the absolute numbers of randomly selected reads mapping from paired samples.

### *De novo* assembly.

Any reads mapping to only the human genome were removed, and the remaining reads (unmapped reads and reads mapping to P. falciparum) were then *de novo* assembled into transcripts using rnaSPAdes with default parameters (v3.10.1) (28). Redundant transcripts were clustered and condensed using CD-HIT (v4.6) and the default sequence identity parameter of 90% (29). The sequence of the longest open reading frame (ORF) from each assembled transcript and its translation were obtained using EMBOSS’s getorf utility (EMBOSS v6.6.0.0), with minimum ORF size set at 500 nucleotides. The resulting peptide sequences were searched using BLASTp (BLAST 2.2.26+) against all ∼4,700 PfEMP1 amino acid sequences from reference genomes and clinical isolates included in the design of the capture array. The protein blast search was repeated against a database of only *var* sequences from the 3D7 reference genome for comparison. Any transcript matching a PfEMP1 amino acid sequence was evaluated for the presence of *var* domains using the VarDom database (18). Sequences for which *var* domains could be annotated with the VarDom database were considered *var* transcripts. The number and length of these transcripts were then evaluated for each sample, from each enrichment method.

### Comparing *de novo*-assembled *var* genes to genome assembly sequences.

Full *var* repertoires are known for a few reference genomes and clinical samples (6, 18). Recently, we sequenced and assembled *var* genes from 12 uncomplicated malaria infections in Malian children with a novel algorithm, providing full genomic *var* repertoires for these clinical infections from a longitudinal study of malaria incidence (19). Samples for RNA extraction were collected and preserved at the same time as those from which DNA was obtained to generate the whole-genome sequence data and full *var* repertoires. As a proof of principle, we compared the *de novo*-assembled transcripts identified as *var* genes with the *var* sequences from the genomic data, which were reconstructed using both PacBio and Illumina reads. Assembled transcripts were compared with the genomic sequences for both coverage (length) and nucleotide sequence identity.

### Quantification of *var* expression.

To compare expression levels estimated from different enrichment methods, and to determine whether we would identify similar predominantly expressed *var* genes, we used the genomic *var* repertoires reconstructed from the genomic data as a reference. An index was created for each sample using the genomic *var* sequences. Using HISAT2, we mapped the reads from each library preparation method using the same index (26) and then determined gene counts with htseq-count (30). Gene counts were converted to transcripts per million (TPMs) to normalize for gene length and library size. TPMs were used to create scatterplots comparing the TPMs from Depletion + poly(A) to Capture. To compare expression levels in the case-control samples with no corresponding genomic reference, for each sample, we generated a reference by *de novo* assembling transcripts using fastq files of combined reads from the Capture and Depletion + poly(A) libraries.

### Data availability.

Sequencing data are available at NCBI through the following accession numbers: SAMN08815609 to SAMN08815630 (longitudinal study samples) and SAMN20286136 to SAMN20286147 (case-control samples).
