# Supplementary figures and images for: Successful Profiling of Plasmodium falciparum var Gene Expression in Clinical Samples via a Custom Capture Array
Source: mSystems. 2021 Nov 30;6(6):e00226-21. doi: 10.1128/mSystems.00226-21 (PMC8631312; doi:10.1128/mSystems.00226-21)

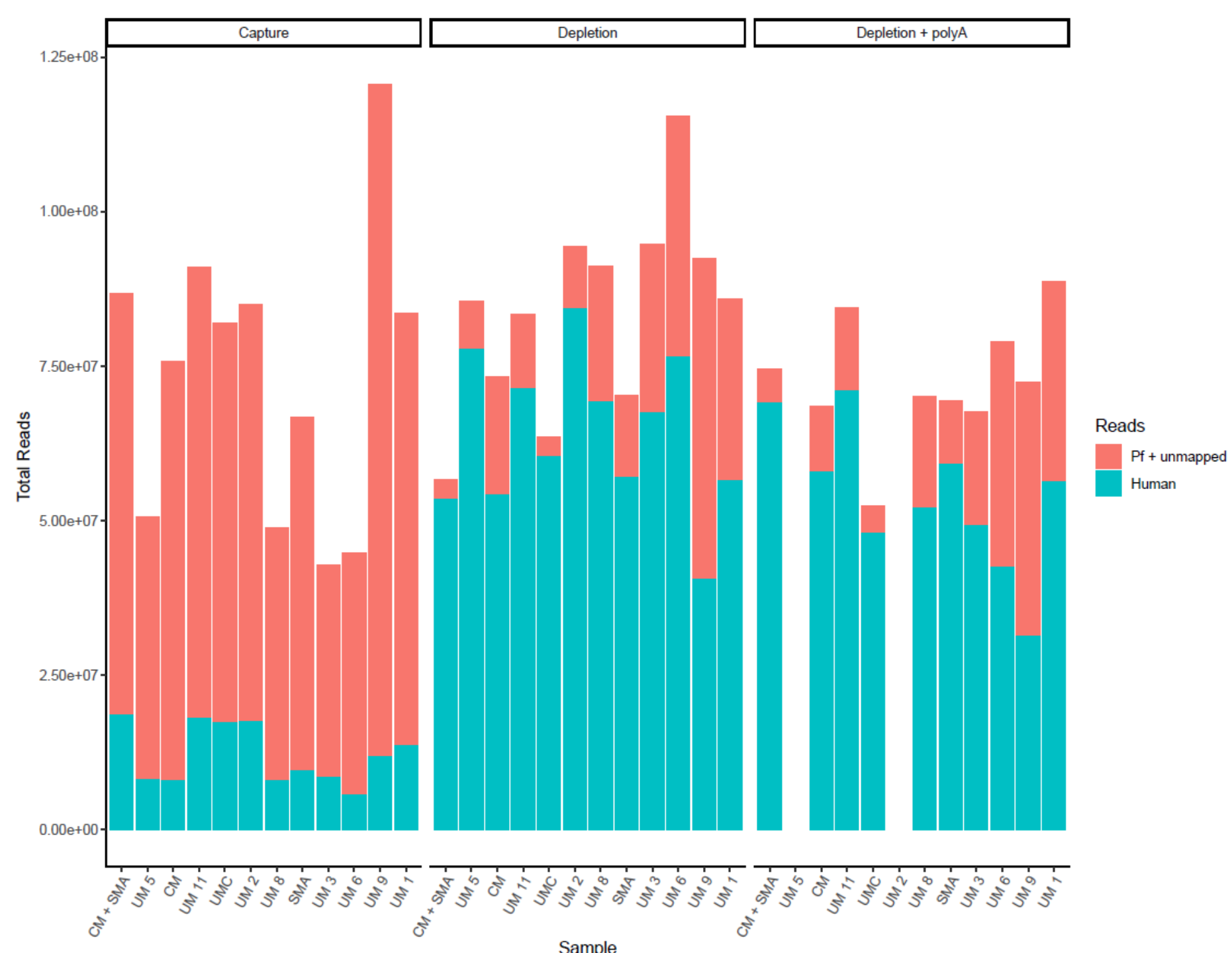

Supplement: FIG S1 [file msystems.00226-21-sf001.pdf]

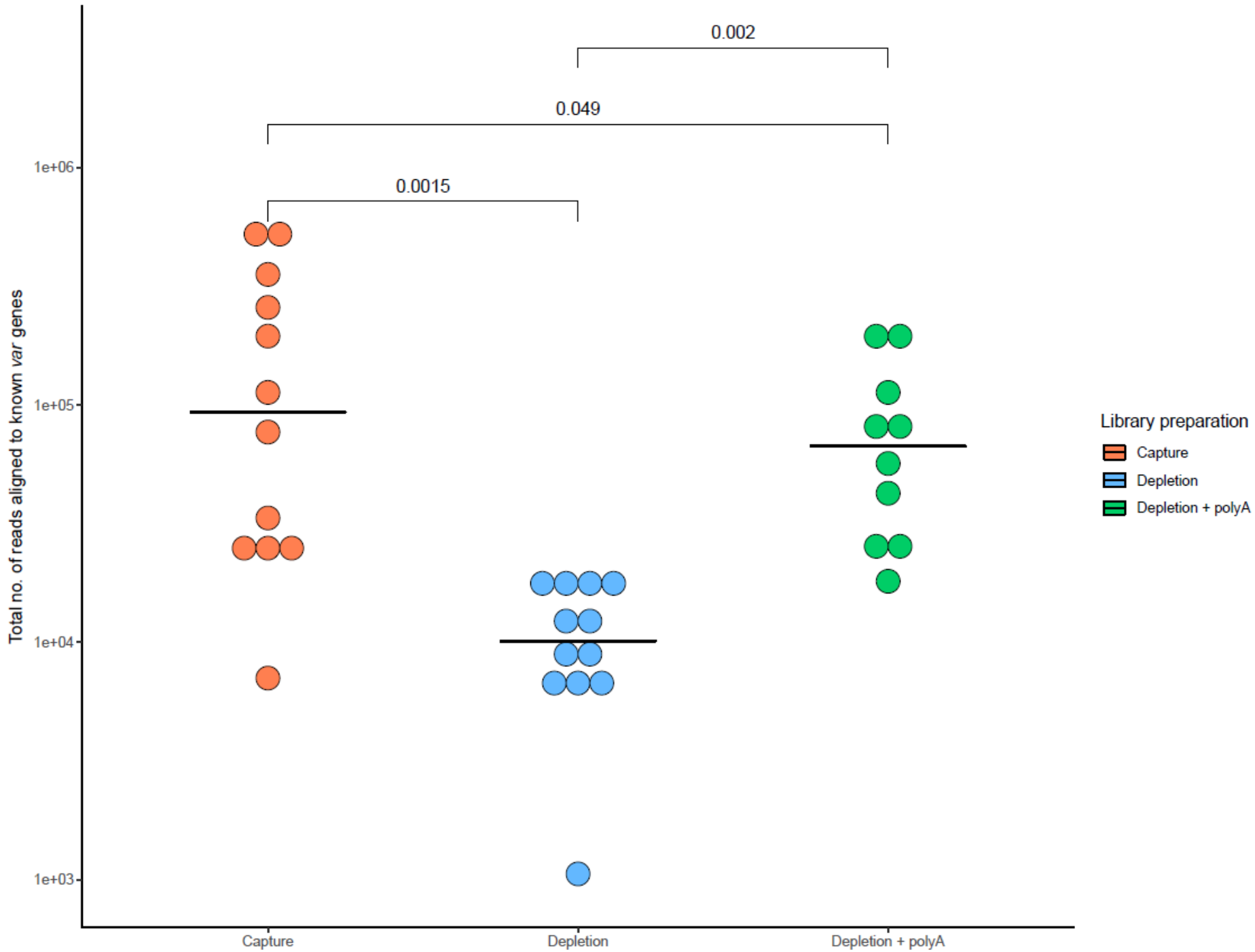

Supplement: FIG S2 [file msystems.00226-21-sf002.pdf]

Supplemental Figure 3

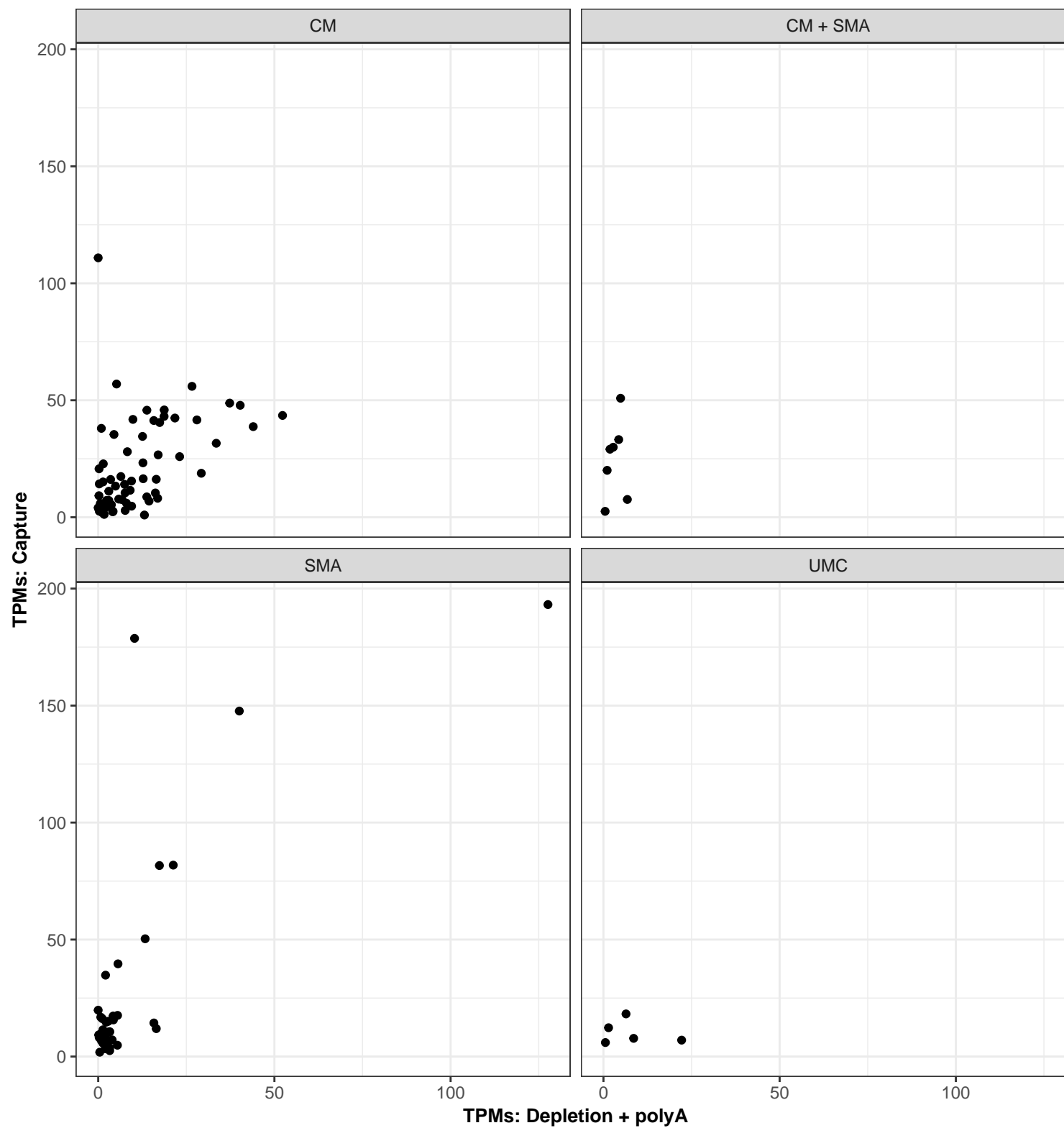

Supplement: FIG S3 [file msystems.00226-21-sf003.pdf]

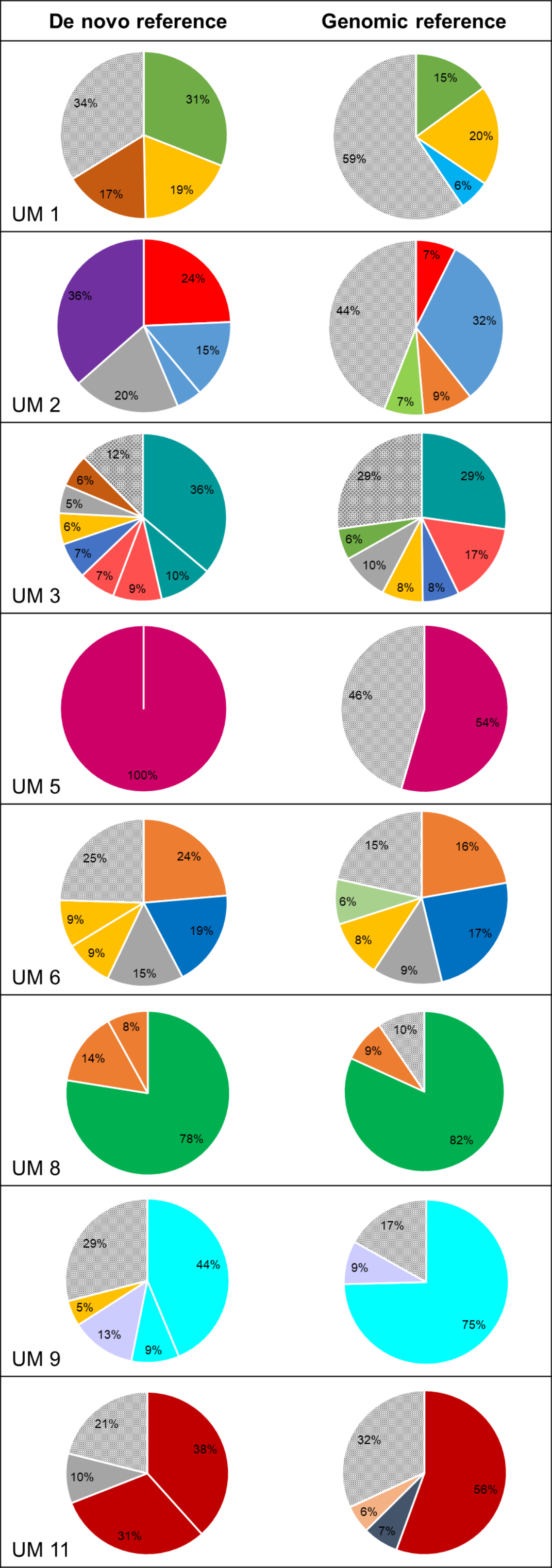

Supplement: FIG S4 [file msystems.00226-21-sf004.pdf]
